# Normal Salt Solution—When to Use It; How to Use It

**Published:** 1902-11

**Authors:** A. P. Heineck

**Affiliations:** Chicago, Surgeon Cook County Hospital; Adjunct Professor of Surgery, College of Physicians and Surgeons, Medical Department University of Illinois


For Texas Medical Journal.

### Normal Salt Solution—When to Use It; How to Use It.

BY A. P. HEINECK, M. D., CHICAGO,
Surgeon Cook County Hospital; Adjunct Professor of Surgery, College of Physicians and Surgeons, Medical Department University of Illinois.

The name, "artificial serum," which is largely used by European authors, we discard. It is confusing. It is inaccurate. It might suggest the existence of a similarity of action and of composition between this substance and the various antitoxins. Such a relation does not exist. Normal salt solution is not antitoxic, is not microbicide (Bosc and Vedel, *Archives re Physiologie,* 1896).

Solutions containing only water and NaCl are called simple normal saline solutions; those containing in addition to the NaCl and water sodium sulphate and other salts, are called compound normal saline solutions.

Normal salt solution is one of our most valuable therapeutic agents. "The use of saline infusions is as specific in its field of usefulness as that of any drug that we possess." Its field of usefulness is being constantly extended. It is a non-toxic agent. It is not noxious to human tissues. It can be obtained in unlimited quantities. In its use, the question of cost does not have to be considered.

"Normal salt solution has never been shown to have any deleterious influence on the tissues" (Hunter Robb). Vaquez examined the blood of animals that had been subjected to repeated saline injections. He says: "The white corpuscles showed no apparent change of form. The normal proportion of the different forms of leucocytes was not altered. No difference in the reaction of the blood elements to the different stains, especially none to eosine, or hemateine, could be detected." "There is no fear of untoward symptoms from the injection of one or even two litres of saline solution at a time" (J. G. Clark).

It does not coagulate albuminous fluid. It does not coagulate blood. Its facility of preparation, its cheapness, its harmlessness, its non-toxicity, its manifold indications, its utility as a life-saving agent, all these and other reasons alike commend its use to the physician. There is no toxic dose, there is only a toxic rapidity of absorption.

Why is the use of so serviceable a medicament as normal saline solution so restricted among the general practitioners? It is because they ignore the indications and the contraindications to its use; it is because they exaggerate to themselves the difficulties and the dangers, if there be any, of the different methods of administering normal saline solution.

Like other therapeutic agents, normal saline solution must be employed (a) with discernment and prudence, (b) must be employed at the proper time, its use never being delayed until all hope of success is futile; (c) must be used in sufficiently large and repeated doses, the quantity to be used, and the frequency of use, varying with the conditions present in each individual case.

The saline solution 7-1000, is the solution of choice to employ in therapeutics. "A 5 per 1000 produces the same physiological action and possesses the same therapeutic effects as a 7 per 1000 solution. However, the physiological effects which we consider the most important, as diuresis, thermic reaction, are not as rapidly produced, nor as marked, when the former solution is used as when the 7-1000 is employed" (Bosc and Vedel).

For transfusion and infusion purposes, normal saline solution has displaced human blood, because the transfusion of blood necessitates a cumbersome and complicated apparatus; because it is difficult to find a donor of blood of strong physique and free from constitutional taint, hence human blood is not at hand when most needed, and cannot be obtained in sufficient quantity. The technique of blood transfusion is far more difficult than that of normal saline solution; during transfusion of blood very painful symptoms are in some instances experienced; after transfusion there may be alarming prostrations, may be hemorrhagic fever; with blood transfusion, there is danger of formation of emboli. Coagulation emboli was often the cause of death when blood transfusion was practiced. The regeneration of the blood corpuscles after transfusion of the blood requires more than double the time required after saline injection. Hunter says, in the *British Medical Journal*, 1899: "Any value which blood transfusion possesses over the infusion of saline solution depends upon the presence of the red corpuscles and their hemoglobin in the general circulation."

To this he adds: "Any advantages that transfusion of red corpuscles may have over saline injections are counter-balanced by the dangers attending the simultaneous injection of the white corpuscles."

In the treatment of hemorrhage, another advantage of saline solutions over blood transfusion is that the hemostatic action of the latter is less than that of the former.

"The injection of normal saline solution is free and safe from all the disadvantages of blood transfusion" (Jno. B. Bullitt). "For transfusion the employment of saline solution has superseded almost entirely the use of blood" (Bryant).

The transfusion into human beings of the blood of inferior animals was discarded because it is not infrequently followed by acute nephritis, suppression of urine, hematuria and uremic symptoms. It imperils life and often leads to a fatal issue. Landois, Ponfick and Panum showed conclusively that the blood of one species cannot be injected without danger into another species.

The use of human serum for transfusion purposes was abandoned because it cannot be obtained in sufficient quantity. Defibrinated blood should not be used, because "the leucocytes so preponderate in it that transfusion of defibrinated blood is an operation, not only dangerous in itself, but one whose practical value by no means compensates for the risks attending it" (Hunter).

Pure water alters the blood corpuscles. It can, however, in emergencies be used for transfusion. Distilled water, however, must not be used, not only because it is difficult to obtain on immediate notice, but especially because when introduced into the blood it is decidedly toxic to the human organism, provoking blood evacuations, causing degenerative changes in the blood corpuscles, and producing hemorrhages. Bosc and Vedel state that as a result of their laboratory experiments they have come to the conclusion that distilled water, when injected, even in small doses, into the blood, has an energetic toxic action. They say that in a general way distilled water should not be used as a vehicle for substances that are to be introduced directly into the blood.

Experiments have been conducted to determine the value of intravenous injections of milk in anemia. These injections have been found to be valueless, to be dangerous.

Dr. Joseph Howe (*Handbook of the Med. Sciences*), in speaking of his experiments upon dogs with the intravenous injection of milk, says that when he had bled seven dogs to a state of syncope, milk was intravenously injected, and not a single recovery took place. The intravenous injection of milk in human individuals is attended with serious accidents.

Various solutions are used by different clinicians. The following are the formulæ most frequently employed:

Hayem's formula:

| | |
|---|---|
| Sodium chlor. | 5.00 |
| Sodium sulph. | 10.00 |
| Boiled filtered water | 1000.00 |

Galvani's formula:

| | |
|---|---|
| Sodium chlor. | 7.50 |
| Sodium bicarb. | 5.00 |
| Boiled filtered water | 1000.00 |

These and other complex mixtures of various salts are recommended, but they take time to prepare, and there is no evidence that the results following their use are better than those that can be obtained by employing simple normal saline solution, which is a solution of common salt in water in the proportion of 7 parts of salt to 1000 of water. It can be prepared with reasonable accuracy, when the urgency of the case demands that no time be lost, by dissolving a teaspoonful of salt in a pint of boiled and filtered water. The water is boiled to sterilize it, and thereby avoid infectious phenomena. It is filtered to remove any foreign bodies present. Foreign bodies, when introduced in the vessels, may lead to emboli formation; when introduced in the tissues they irritate them. Foreign bodies may block the lumen of the needle or cannula.

I have used various solutions and clinical observations led me to believe that the other solutions possess no advantage over simple normal salt solution. There is only an infinitesimal proportion of sodium sulphate in normal blood. Its addition to simple normal saline solution is of no discernible advantage; according to Mezet, it appears to be injurious to the integrity of the blood corpuscles. Simple normal saline solution is the most easily prepared, the most inoffensive and the most widely used of the various saline solutions.

Common salt and water are found in every household. Simple normal saline solution, according to Bosc and Vedel, produces the maximum physiological effects—most profuse diaphoresis and diuresis. It is the solution exclusively employed in the Cook County Hospital.

USE OF NORMAL SALT SOLUTION.

1. As an inexpensive cleansing spray in chronic hypertrophic rhinitis (Morris J. Asch). As a gargle (Geo. A. Leland).

2. In diphtheria, irrigation of throat with hot normal saline solution gives much relief. Its use can be repeated every four hours. Irrigation removes all the loose membrane (J. H. McCollom).

It being definitely understood that its use in this connection is only as a palliative and as a cleansing agent, antitoxin being the specific remedy in this infection.

3. In skin grafting and to douche skin grafts previous to transplanting them. Antiseptics exert a deleterious action on living cells, and when used in skin grafting endanger the success of the operation. They impair the vitality of the grafts. Antiseptics must be thoroughly removed by prolonged douching with sterilized normal saline solution, from areas that are to be skin grafted.

"In bone grafting, the bone to be engrafted should be rendered thoroughly aseptic and should be kept in a sterilized salt solution at a temperature of 105°-110° F." (Wharton).

4. As an irrigating fluid for wounds. When used for this purpose it must be sterilized. When used as an irrigating fluid for wounds, it should be warm. Cold solutions chill the patient. Saline solution used for this purpose should have been boiled half an hour. Normal salt solution can be boiled for sterilization without changing its composition. "Saline solution has a positive antiseptic action" (Tavel). It is less irritating to the hands of the surgeon than the repeated employment of solutions of antiseptics. It does not irritate the tissues. It does not impair their physiological properties. Can be used for irrigation of the uterus. Used in abdominal surgery to wash out septic material. Owing to the great absorptive powers of the peritoneum, solutions containing germicidal drugs, when used in abdominal surgery, in addition to their local irritating action, can produce systemic disturbance from the absorption into the system of the chemic irritants which they contain. "In abdominal surgery, warm sterilized salt solution is the best fluid to employ for irrigating the serous cavity or for washing out sponges, swabs or cloths" (Rose and Carless). Valuable for gastric lavage. For lavage of the urinary bladder.

5. In diarrheal affections. It is also of great value in gastrointestinal infections of infancy. Injections of normal saline solution, whether intravenous, subcutaneous or rectal, raise the arterial pressure and hydrate the tissues. They quiet the intense thirst present in these conditions. "Emptiness of the circulatory cavities is the cause of the collapse in cholera" (B. J. Richardson). After laryngeal intubation for diphtheria, to quench the patient's thirst give massive injection of normal saline solution. It is harmless. Enough of the solution is retained to allay thirst. After intubation, fluids are swallowed with difficulty. Trickling down the trachea they excite cough and may provoke deglutition pneumonia.

In the following affections the use of normal saline solution will be found very serviceable:

(a) Cholera asiatica (its use in this disease is sanctioned by the best authorities). "Owing to the profuse serous discharges the blood becomes concentrated and absorption takes place rapidly from the lymph spaces. To meet this, intravenous injections have been practiced. This is really a valuable method, thoroughly physiological, and should be tried in all severe cases" (Osler).

(b) Cholera nostras.

(c) Acute enteritis.

(d) Dysentery.

(e) Gastro-intestinal intoxication of nurslings.

In profuse diarrheas, the sunken eyes of the patients show how wanting in fluid the tissues are.

6. In the anemic form of asphyxia neonatorum. "The infant is suffering from shock, and the measures which suggest themselves are similar to those we would resort to were we dealing with an adult. Heat to the surface, injection into the rectum of a pint of hot (115° F.) saline solution, the instillation into the mouth of ten drops or so of brandy, such are the primary measures of utility" (Grandin and Jarman, *Practical Obstetrics*).

7. In surgical and puerperal infections. In these conditions the use of normal saline solution is an adjuvant measure, to which must always be associated the habitual therapeutics of these infections. Results following its use in these conditions are often extraordinary. "The subcutaneous employment of large doses of normal saline solution can be resorted to with advantage in infectious states, when symptoms of marked depression are present." The use of normal saline solution raises the arterial tension. This increase of arterial pressure causes increased diuresis. This increased diuresis is attended by an increased elimination of toxins. Tuffier has practiced intravenous injections both for hemorrhage and for toxemia in fifty cases, and found the first effect was to restore the normal arterial pressure and to diminish the frequency of the heart's action; the second, to cause diuresis. He says that normal saline solution is of special value in surgical and obstetrical infections.

Other authors that highly recommend its use in these conditions are Lejars, Delbet, Duret, Jayle, etc. "In puerperal septicemia, they (saline injections) aid the patient to better withstand the noxious influence of the bacterial toxins" (Michaux, Segond).

Infections due to the bacillus coli communis are retarded and combatted by injections of normal salt solution (Bosc, Vedel. Manquat). "In septicemia, we endeavor to dilute the poison and eliminate it through the general secretory organs. We do this by means of enormous quantities of normal salt solution injected into the tissues. In sapremia, the use of saline solution, by transfusion or by venous transfusion, dilutes the poison, and stimulates the heart, skin and kidneys into activity" (Da Costa).

There is a relation existing between the infectious states and leucocytosis. In infectious conditions, there is an increase in the number of leucocytes present in the blood. With the advent of convalescence the number of leucocytes present in the blood diminishes.

The use of saline solution, when employed in pathological states, is most always followed by a diminution in the number of leucocytes. "The marked leucocytosis present in most infections diminishes rapidly after the use of injections of normal saline solution" (André Claisse). The same author reports experiments conducted on animals, in which the use of injections of normal saline solution was followed by a marked diminution in the number of leucocytes.

Among the many effects produced by normal saline solution are increased force and regularity of pulse, except in cases where the arhythmia is due to a cardiac lesion; it stimulates the cardiac ganglia, dyspnea is relieved; increased renal activity, amount of urea excreted is increased. Diarrhea is often provoked; increased glandular activity, production of saliva is increased; tongue becomes moist and thirst disappears; increased activity of sweat glands; skin becomes moist a few hours after the injection; elevation of temperature, whether patient be hypo- or hyper-pyrexic; following the injections there is a multiplication of hematoblasts. Injections of salt solution increase arterial tension and act as a "whip" to all emunctories. "They may also stimulate phagocytosis and may be tried in serious cases of broncho-pneumonia" (Anders).

Readers wishing to get further information as to value of saline solution in surgical infectious states are referred to Lejars, *Soc. de Biologie,* 1896.

8. In malignant forms of syphilis that fail to respond to the usual anti-syphilitic treatment. "How does this medication (normal saline solution) act? Is it by exciting diuresis? Is it owing to a depurative action, resulting from the renal hyperactivity which it excites, this renal hyperactivity causing an elimination of toxins? Is it by provoking leucocytal activity and thereby increasing the individual's resistance against the virus? The diuresis following the injections is most abundant when the febrile reaction consecutive to the injection is most marked."

9. In uremic poisoning. "In case of insufficient excretion through the kidneys, stimulation of this function can usually be accomplished by injections of normal saline solution into the bowel. From one to three quarts being so employed once or twice daily" (Bullitt).

The amount of urine excreted by the kidneys being dependent upon the blood pressure in the kidney (Woolsey), increasing that blood pressure by increasing the amount of circulating fluid increases the urinary flow. Saline injections, be they intravenous or rectal, markedly increase the quantity of urine (Bovee). They considerably augment the amount of urea excreted (Volt, Rabuteau).

The amount of sodium chloride excreted by the kidneys is enormously increased. During my internship at the Cook County Hospital the routine treatment employed in uremic poisoning was the following. We found it very satisfactory, and through its use many apparently hopeless cases recovered. I used it personally in the services of Professor A. R. Edwards and Professor G. Butler, and have great faith in its value:

1. Milk diet and an abundance of water.
2. Hydragogue cathartics. Jalap and salines.
3. Alcohol sweat daily, preceded either by a hypodermic injection of pilocarpine nitrat, gr. 1-6, or strychninæ sulph., gr. 1-25, nitro-glycerine, gr. 1-30, according to the condition of the patient's heart or lungs.
4. Enema of Oj. of normal saline solution, t. i. d., to be retained. Tube inserted high up. Barre has obtained excellent results in cases of uremia by injecting intravenously in uremic patients a quantity of saline solution equal to the amount of blood simultaneously withdrawn.

10. Puerperal eclampsia. Porak reports eight cases in which he used saline solution. Six recoveries. Audebert also recommends its use in this affection.

11. Before operative procedures, when patients are anemic, either from repeated small hemorrhages or from one profuse hemorrhage. "When in a patient weakened by large losses of blood or whose general conditions is bad, it becomes necessary to perform an obstetrical operation (the author might have said any operation), it is wise to make either an intravenous normal salt solution or one or more subcutaneous injections. The patient will thus be enabled to better withstand the operative shock, and to offer better resistence to any hemorrhage that may occur" (P. Millet).

In collapse while patient is being operated on. Here it is used as a cardiac stimulant. Intravenous or subcutaneous injections can be performed while patient is being operated on. After operations, it quenches thirst; it contributes to the restoration of the blood volume; it lessens shock. Dr. Kelley (Baltimore) has employed sub-mammary infusion in forty-one out of two hundred and twenty-five cases of abdominal surgery. Of these forty-one cases none of them suffered with as much as a cellulitis. Hunter Robb says: "I am convinced that the use of salt solution undoubtedly diminishes the shock which generally follows a serious abdominal operation."

Normal saline solution, when administered after operations, lessens the tendency to suppression of urine. It is valuable in aiding in reaction from the shock (death from shock after exhaustive hemorrhage, directly after the patient leaves the operating table, is by no means rare) incident to the anesthetic and to the operation.

After post-partum hemorrhage and operations in which there has been much hemorrhage, I always prescribe an enema of one quart of warm saline solution (enema to be retained), to be repeated every four hours until three have been taken.

I have found this to be a very valuable procedure. If condition is serious, I order the enemata to be repeated hourly until the quality of the pulse improves. From two to four ounces of brandy can with advantage be added to the first enema.

12. In diabetic coma. Transfusion of saline solution has proved of service to prolong life.

13. In the treatment of electric burns, use normal saline solution fomentations. Gauze saturated with normal salt solution is also valuable in the treatment of ordinary burns.

14. In tapping or withdrawing fluid from a chronic hydrocephalus or from a meningocele, the too rapid withdrawal of the fluid may cause alarming symptoms. If it does, inject in the ventricular or in the subarachnoidean space some normal salt solution.

15. To quench the thirst in affections in which no food must be given per mouth, as in Professor Ochsner's starvation treatment of appendicitis.

To quench thirst after laryngeal intubation give massive rectal injections of normal saline solution. After intubation liquids are swallowed with difficulty; trickling down the trachea, they excite cough and may provoke deglutition—pneumonia.

16. In some forms of vegetable poisoning, as in toadstool poisoning (Noyer, *Presse Medicale,* Sept. 30, 1899).

17. In gonorrheal prostatitis, rectal irrigations are of value. White and Martin direct that they be used as follows: "A quart of seven-tenths of 1 per cent. salt solution (110-115° F.) is used. The injection pipe is introduced into the anus and its end tilted upward and forward so that the stream when it is turned on shall flow directly on the prostatic tumor as it bulges into the rectum. The exit pipe allows the fluid to flow away as fast as it enters the bowel. This treatment should be repeated two or three times daily."

18. In shock, or in collapse following large hemorrhages, we have no other therapeutic agent that will give results comparable in value to normal saline solution. "No patient should be allowed to suffer from symptoms of shock or depression without its employment." Exact amount given being governed by the reaction of the patient. If after a few hours symptoms of depression again supervene, the injection should be repeated. Inject several pints to begin with. Inject fluid at a temperature of 105-110° F.

In shock normal salt solution is valuable to stimulate the heart and arteries, so that the blood which has accumulated and stagnates, especially in the large abdominal vessels, may again be put in active circulation. The increased arterial tension furnishes more nutrient blood to the heart through the coronary arteries, thereby stimulating and energizing the action of the heart. As the right heart feels the pressure of the injected fluid, the heart beats become lengthened and more forcible and the blood pressure in the peripheral arteries rises.

Bruntun says that shock is mainly due to paralysis of the heart and vaso-motor paralysis of the abdominal vessels. The sudden dilatation of the abdominal vessels causes symptoms similar to that of sudden or profuse hemorrhage. The blood pressure falls, the remote arterioles contract, the heart falters. May Robson, in the *British Medical Journal*, 1893, describes cases in which infusion of several pints of normal saline solution into the circulation warded off impending death in cases of intense shock after operation.

Symptoms of shock are chiefly due to the accumulation of blood in the abdominal vessels, where, for the time being, it is useless, and to its withdrawal from other parts, leaving the head and extremities in a state of anemia. The increase of the circulating fluid following saline injections overcomes this anemia of the vital centers and insures their functionating.

After abdominal operation leave a pint or more of normal salt solution in the abdomen. It minimizes shock. It is absorbed with great rapidity after the operation, if the patient is placed with his head lower than his feet, because in this position the saline fluid gravitates to the diaphragmatic region, where absorption is very rapid.

19. To combat the effects of hemorrhage normal saline solution is the agent par excellence. The more general and frequent use of saline injections in all cases of acute anemia from hemorrhage and of profound shock, that do not respond to ordinary stimulation, cannot be too strongly urged. "Many a life is now lost in private practice after severe post-partal or other bleeding has been controlled, because the family doctor has not had the nerve, or the experience, or the appliances thought necessary to accomplish infusion, and the remaining amount of circulating medium being not sufficient, the patient succumbs" (Dawbarn).

"A patient should never be abandoned as dead from hemorrhage unless copious saline infusion has been performed, for surprising results have followed when it had been administered even in apparently hopeless patients" (Morton).

Its use is indicated in all forms of hemorrhage, be the hemorrhage traumatic, operative, post-operative, post-abortum or post-partum. The practitioner will find it of value to combat the anemia resulting from intestinal hemorrhage, as in typhoid fever; from hematemesis, as in ulcer of the stomach; from uterine hemorrhage, as in fibroma, cancer uteri; from hemorrhage due to ruptured varicose veins; from concealed hemorrhages, as in ruptured extra-uterine pregnancy.

"In hemorrhage, the real cause of death is a mechanical one and consists in the loss of volume of the circulating fluid, which must within certain limits be proportional to the capacity of the vascular system" (Pye-Smith).

Schwartz, in 1881, injected in rabbits a quantity of saline solution equal to half their blood mass. These massive injections did not determine any accidents. He demonstrated that in rabbits, the loss of two-thirds of their blood is not productive of death, if these (the two-thirds) are replaced by an equal quantity of salt water.

Injections of normal saline solution increase the volume of the blood and lessen its specific gravity. This abundant adjunction of liquid to the circulating fluid is of value after hemorrhage, because by restoring the blood mass, it re-establishes the arterial pressure necessary to stimulate the centers in the medulla oblongata. With the disappearance of the anemia of the medulla oblongata, vital functions return. "The immediate source of danger from sudden loss of blood is the fall of blood pressure to a point where the circulation cannot be maintained" (Hunter, Reilly).

Vascular tension is the useful element in transfusion. The heart does not contract, does not perform its function, unless a sufficient quantity of fluid is poured into its cavities. "One of the most important causes of the immediate serious symptoms resulting from hemorrhage is the diminution in amount of the circulating fluid rather than the changes in its quality" (C. S. Bacon).

It is deficiency of fluid. It is the disturbed relation between the calibre of the vessels and the quantity of blood contained in them that causes death in hemorrhage.

"After hemorrhage, the dominating indication is to quickly restore the volume of the circulating fluid to that point which will make possible the continual action of those forces by which its continual flow through the vascular channel is carried on" (Pilcher).

The dominating indication is best met by the injection of normal saline solution. Under its influence the lowered blood pressure due to profuse hemorrhage is rapidly brought to normal; the pulse becomes palpable; respiratory action is stimulated; warmth returns to the periphery. The tongue becomes moist; the voice becomes louder.

Normal saline injections have an hemostatic action (Hayem).

The coagulating properties which they possess prevent the increased arterial pressure which follows their use to be attended by a renewal of the hemorrhage. This hemostatic action of normal saline solution is due to its exciting (a) vaso-motor constriction of the ruptured arterioles; (b) to the fact that it activates the precipitation of the hematoblasts at the site of hemorrhage, thus forming centers of clot formations.

When combatting the effects of hemorrhage by the infusion of normal saline solution, the bleeding points, when accessible, must be secured. When the source of hemorrhage is not accessible, as in the intestinal hemorrhage of typhoid fever, we must begin with subcutaneous injections of small quantities, say 200 to 300 cubic centimetres. Later we can employ larger doses. Thompson (*Medical Record,* November 11, 1899) recommends subcutaneous injections of normal salt solution in the treatment of hemorrhages of typhoid fever. He injects the solution in the lumbar region between the crest of the ilium and twelfth rib.

There are three principal routes for introducing normal saline solution into the human system: The rectal route, the subcutaneous and the intravenous. The use of salt solution by mouth is not practicable; it excites nausea, it may provoke vomiting and it is impossible to ingest sufficient quantities that way. The intra-arterial route is condemned by most of all the authorities. Its use has been followed by serious accidents, such as gangrene. Permanent dilatation of the walls of the artery and even sloughing of the soft parts have followed the employment of the intra-arterial route. The injection of normal saline solution through the abdominal wall into the peritoneal cavity carries with it the danger of wounding the intestines. I cannot recommend it. Some clinicians have injected the solution in the pleural cavities. Their results were not encouraging. A method of administering normal salt solution that is gaining favor consists in filling the peritoneal cavity full of the solution before the belly-wall sutures are tied.

When the condition of the patient is not critical, the rectal route is employed. Lepine says: "The therapeutic effect of the rectal injections is precisely the same as that of the subcutaneous injections." Eitz, Bolognesi and Pauchet have noticed that the rectum is capable of absorbing large quantities of salt water and that these enemata favor diuresis and diaphoresis. It is painless to the patient. It does not require the presence of the physician. It can be administered by nurse or patient's attendant. The solution employed does not need to be sterilized. The possibility of local infection following the use of the rectal route does not exist. With a careless operator, local infection may follow the employment of the subcutaneous or intravenous methods.

To practice a rectal injection of normal saline solution are needed (a) a three-quart fountain syringe; (b) a long rectal tube. In the absence of a rectal tube a rectal canula can be used. "For voluminous rectal injections of normal saline solution to be most effectual, the tube must penetrate the sigmoid flexure and command the absorptive powers of the descending, the transverse and the ascending colon; therefore, a long tube must be used and the injection pushed to tension limits" (McNeil). (c) Some plain water; (d) an abundance of normal saline solution. Injections must be repeated as often as the patient's condition demands it.

Proceed as follows:

(a) Give patient a cleansing enema. After voidance of this (b) elevate foot of bed. If that is not convenient, patient's buttocks must be elevated. The bed is elevated so that the saline fluid will pass into the bowel by gravitation. It quickly gets into the circulation, owing to the large absorbing surface.

(c) Patient is to lie on right side. Body to be arched.

(d) Have your solution at the temperature of the body. It has been shown by experiments that the absorptive property of the rectal mucosa is most active when substances injected have the body temperature. Cold solutions must not be used. Cold solutions occasion chills. For voluminous injections, a well-lubricated rectal tube is useful.

(e) In inserting the rectal canula or nozzle bear in mind that the rectum at its lower portion for the extent of from three to four cm. comes from above down and from behind forward.

(f) In some conditions it may be necessary to press a pad against anus, or buttocks can be pressed together so as to have the solution retained. If the intestinal susceptibility prevent the retention of the normal saline enemata, insert tube high up and make injection under low pressure. The solution must not be injected too forcibly or too quickly. If it is, it will excite peristaltic action. About one pint should be injected at a time. Injections can be repeated every four hours if needed.

The subcutaneous method: Subcutaneous injections, though slower than intravenous, are devoid of danger when done with a proper technique and aseptically. They may be superficial—that is, under the skin. They may be deep in the muscular tissues. In using the subcutaneous route (a) solution must be absolutely sterile, (b) the strictest asepsis must be observed in all manipulations. Proceed as follows:

1. Select a region where the cellular tissue is loose and easily distensible, as the axilla, the buttocks, the subscapular region, the abdominal wall, etc. The infraclavicular, the retro-mammary and retro-trochantern regions can be selected.

2. Site of injection must be sterilized: (a) scrub with soap and water; (b) wash with alcohol; (c) wash with HgCl, 1-2000; (d) irrigate with sterile water.

3. Needle, tube and receptacle containing solution, as well as the solution itself, must be sterile.

4. The temperature of the solution should be from 105 degrees F. to 110 degrees F. Abont 700 cu. cm. can be safely injected through one puncture. Experiments have shown that the absorption is most rapid when the arterial tension is low (Feilhenfeld). You can inject simultaneously at more than one point so that absorption will go on more rapidly.

5. In inserting needle, be careful not to wound any superficial veins. Introduce needle while fluid is flowing, so as to avoid the introduction of air in the tissues. Usually simple elevation of the reservoir containing the saline solution is sufficient to force the fluid into the loose cellular tissues. "In the subcutaneous method the solution is allowed by gravitation to enter the circulation of the patient" (Stepp).

6. After from 500 to 700 grammes of solution have been injected through a single puncture the needle is removed. The small cutaneous wound made by the needle is to be occulated by a few drops of collodion and some sterile absorbent cotton. Dressings are to be applied to wound. These dressings are to be kept in position by bandages. The absorption of the injected fluid is hastened by massaging the tumor mass with the hand. In healthy individuals the edema attending the subcutaneous injection of the saline solution disappears in a few hours. In cardiopaths and nephritics it takes at times from three to five days for resorption to take place.

Advantages of subcutaneous method over intravenous method:

1. The patient's and his parent's consent to subcutaneous injections is more easily obtained than for intravenous injections. The procedure not necessitating an incision, is not considered an operation. The injections are more easily repeated.

2. No anesthetizing of area to be incised, no cutting, no stitching of wound are necessary. There is no danger of introducing air into the circulation. The fluid is slowly taken into the general circulation, thus avoiding the danger of sudden increase in blood pressure.

3. Technique is simpler. Fewer instruments needed. Douche bag or syringe, piece of tubing, hollow needle from the aspirating case, or from the hypodermic case, are all the instruments needed.

There are only a few objections to the subcutaneous route. They are the pain incident to puncture and to repetition of punctures. The pain is slight. There is some pain due to distension of the skin. This, however, does not last long. The possibility of local infection and abscess formation does not exist when surgical cleanliness is observed, and when overdistension of the skin is not excessive, massaging the site of injection by hastening the absorption of the injected fluid will prevent the evil effects of overdistension. Infection following subcutaneous or intravenous injection of normal saline solution is evidence of a negligence on the part of the surgeon.

One of the great advantages of subcutaneous injections is that they, owing to the simplicity of their technique, can be given by the nurse.

The intravenous method:

In urgent cases, owing to its greater rapidity of action. the intravenous route is preferable.

Objections to the use of the intravenous route:

1. When patient has lost much blood the veins are not full and are difficult of access.

2. Lumen of vessels may be so small that neither the infusion canula nor the smallest aspirating needle can be inserted (J. G. Clark).

3. Vein is often difficult to find in children, in women and in obese individuals.

4. Cardiac lesions and myocardiac weakness should always make us prefer the subcutaneous and rectal route. "By intravenous transfusion cardiac paralysis may be induced through over-distension" (Bovee).

5. Danger of producing an acute pulmonary edema. "Generalized pulmonary edema is one of the most frequent accidents of intravenous injections."

6. The procedure is not painless and requires careful asepsis. "Cases of phlebitis and embolism following the intravenous method are not uncommon" (Thienot).

Too rapid intravenous injection of normal saline solution may cause cardiac distension, pulmonary disturbances, as pneumonia, edema of lungs, etc. Formeaux noted engorgement of spleen and liver, with marked pain in the latter, in one case. Pleural, peritoneal and subarachnoid effusions have been noted by other observers.

In adults the intravenous injection of 1500 grammes in about fifteen minutes does not determine any symptoms of intolerance. In children, be the route the rectal, the subcutaneous or the intravenous, the dose is to be proportioned to the age and weight of the patient. The condition of the patient's pulse will tell us when enough fluid has been injected. This, however, does not hold good in sepsis.

The fact that its results are more rapid and that by it heat is brought directly to the cardiac and arterial ganglia, is what commends the intravenous method to many clinicians.

Technique of intravenous method: The following instruments are needed:

1. A scalpel; two dissecting forceps to elevate tissues and aid in isolating veins; a constricting band; an aspirating needle, a hypodermic needle can do, a metal or glass canula, the end of which is bulbous, blunt and bevelled and of such a caliber that it can easily be tied into the selected vein is preferable to either; a large funnel or recipient provided at its lower extremity with a rubber tube 1 m., 50 cm. long, to which is adapted the aspirating needle or a large hypodermic needle; surgical needles and silkworm gut to suture incision once transfusion is completed; silk or catgut to ligate distal and proximal ends of veins; two hemostatic forceps to check cutaneous hemorrhage.

2. Any large vein can be selected. Veins at the bend of the elbow are the ones usually chosen. If veins of arm are chosen, right arm is preferable, because its veins are a trifle more prominent. If vein of elbow bend be selected, apply constrictory band midway between the elbow and shoulder joints sufficient to interrupt the venous return, but not so tight as to impede the arterial flow. The median cephalic is preferable to the median baselic owing to its greater distance from brachial artery and posterior relation of the external nerves. The internal saphenous vein can be selected. It is easily accessible. It is a good vein to select. Its caliber is larger than that of the veins of the elbow. It rests upon a bony plane and is easy to locate in fatty subjects. The introduction of a little air, though of slight importance when occurring at such a distance from the heart, does not offer much danger.

3. All instruments above mentioned must be sterile. Site of injection must be disinfected; surgeon's and assistant's hands must be surgically clean. Anesthetize line of incision either with ethyl chloride or by intra and subcutaneous injections of 1 per cent. solution of cocaine. Schleich's solution can be used. If forearm is selected, before making incision place it in position of supination.

4. A constricting band is tied about extremity above the selected site for operation, so as to lessen the upward diffusion of the anesthetic and so as to congest the veins and make them stand out. This is to be removed as soon as needle has been introduced in vein. Or the limb may be made to assume a dependent position over the side of the table or bed. If this does not make them prominent (because of non-action of the heart or small amount of circulating medium) rubbing the parts in the direction of the arterial supply will generally suffice.

5. Expose vein selected. It is the only way to introduce the needle with precision and accuracy. Introduction of the needle into the vein through the skin is a blind, unreliable, unscientific procedure. Vein is to be opened between two ligatures. Glass tube or needle is to be introduced in vein while the fluid is escaping, being careful that fluid penetrates vein and not sheath. Canula is thrust upwards in proximal end of vein—that is, in the direction of the blood current, for one-half to three-quarters of an inch, and secured in position by tying down the upper ligature so as to bring the vein wall tightly about the canula. All the air must be carefully expelled from the tube and needle before it is introduced in vein by allowing fluid to run through them. Solution should have a temperature of about 105 degrees to 115 degrees F. Receptacle is raised 1.5 meter above level of the bed. This allows the liquid to flow at the rate of 1500 grammes of liquid in twenty-five to thirty minutes, the rapidity of the flow being controlled by the height of the receptacle and by the caliber of the needle. The higher the receptacle, the more rapid the flow; the larger the caliber of the needle, the greater the volume of the fluid flowing through it in a definite space of time. The patient should always be watched for some hours after transfusion and if the pulse again fails the transfusion must be repeated.

Quite often it is necessary to repeat these intravenous injections. The same vein can be used for successive operations. It is then to be incised a few centimetres above the first incision.

1 Journ. Am. Med. Assn., 1898.
2. Bosc and Vedel, Archives de Phys., 1896.
3. André Claisse, Rev. de Chir., 1896.
4. Bosc and Vedel, Soc. de Biologie, 1896.
5. Boulengier, Presse Medicale Belge, 1899.
6. American Text-book of Surgery.
7. Dalche, Gazette des Hospitaux, 1897.
8. André Claisse, Soc. de Biologie, 1896.
9. Victor Augagneur, Bull. de la Societe de Dermatologie et de Syphilis.

10. Loomis and Thompson's System of Medicine.
11. J. C. Clark, Am. Journal of Obstetrics.
12. Dawbarn, Medical Record, 1892.
13. Morton, Therapeutics Gazette, 1896.
14. Vaquez, Soc. de Biologie, 1896.
15. C. S. Bacon, Medicine, 1897.
16. Lewis S. Pilcher, Annals of Surgery, 1892.
17. Haymen.
18. Stepp, Clev. Med. Gazette, 1897.
19. Thos. F. Reilly, Trans. N. Y. Med. St. Assn., 1898.

---

For Texas Medical Journal.

